# Non-invasive absolute measurement of leaf water content using terahertz quantum cascade lasers

**DOI:** 10.1186/s13007-017-0197-z

**Published:** 2017-06-17

**Authors:** Lorenzo Baldacci, Mario Pagano, Luca Masini, Alessandra Toncelli, Giorgio Carelli, Paolo Storchi, Alessandro Tredicucci

**Affiliations:** 1 NEST, CNR Istituto Nanoscienze and Scuola Normale Superiore, Piazza San Silvestro 12, 56127 Pisa, Italy; 2 Consiglio per la ricerca in agricoltura e l’analisi dell’economia agraria, Centro di ricerca per la Viticoltura e l’Enologia, Viale Santa Margherita 80, 52100 Arezzo, Italy; 3 Dipartimento di Fisica, Università di Pisa, Largo Pontecorvo 3, 56127 Pisa, Italy; 4 NEST, CNR Istituto Nanoscienze and Dipartimento di Fisica, Università di Pisa, Largo Pontecorvo 3, 56127 Pisa, Italy

**Keywords:** Terahertz quantum cascade laser, Water content, Draught stress, *Vitis vinifera* L.

## Abstract

**Background:**

Plant water resource management is one of the main future challenges to fight recent climatic changes. The knowledge of the plant water content could be indispensable for water saving strategies. Terahertz spectroscopic techniques are particularly promising as a non-invasive tool for measuring leaf water content, thanks to the high predominance of the water contribution to the total leaf absorption. Terahertz quantum cascade lasers (THz QCL) are one of the most successful sources of THz radiation.

**Results:**

Here we present a new method which improves the precision of THz techniques by combining a transmission measurement performed using a THz QCL source, with simple pictures of leaves taken by an optical camera. As a proof of principle, we performed transmission measurements on six plants of *Vitis vinifera* L. (cv “Colorino”). We found a linear law which relates the leaf water mass to the product between the leaf optical depth in the THz and the projected area. Results are in optimal agreement with the proposed law, which reproduces the experimental data with 95% accuracy.

**Conclusions:**

This method may overcome the issues related to intra-variety heterogeneities and retrieve the leaf water mass in a fast, simple, and non-invasive way. In the future this technique could highlight different behaviours in preserving the water status during drought stress.

## Background

There is an increasing need to improve the knowledge of the water resources of plant varieties, owing to the variable rainfall after climate change, in order to assess the need for irrigation [1]. In this perspective, leaves are essential organs for the water balance [2]. Leaf morphology comes from a long evolutionary process of a polyhedral anatomical structure in which the veins are definitely at the heart of this organic evolution [3]. This structure is an essential tool for the mechanical support of the anatomical organization but it also plays a crucial role in the photosynthesis efficiency [4, 5] and in the consumption of water. Furthermore, the capillary branching of the veins in the leaf allows better cooling [6] with potential benefits for the photosynthesis performance [7]. Recent studies show that the vein density per unit area [8] and the thickness of the mesophyll [9] may be involved in the efficiency of hydraulic performance. A decreasing hydraulic functionality, due to water stress or to a limited vein network density, can involve a loss of production. The leaf water potential measurement, defined as the measure of the free energy per unit volume of water (J $\text {m}^{-3}$) [10], explains the level of plant drought stress. The first water potential measurement came in 1960 with two basic instruments: the thermocouple psychrometer and the Scholander pressure chamber. Leaf water potential measured with the Scholander pressure bomb [11] is considered the reference method by plant physiologists. However, this device needs a destructive sampling of the leaf and presents different operating limits: it requires a cylinder with a propellant of nitrogen (or compressed air), cumbersome instrumentation, long operating times and the measurements cannot be automated. The thermocouple psychrometer, instead, is based on the principle that the relative vapour pressure of a solution or piece of plant material is related to its water potential. The requirement of tight temperature control restricted the use of uncompensated thermocouple psychrometers in field studies to situations where good laboratory facilities were at hand. The water content of plants is another basic parameter which describes the plant water deficit and is commonly determined by weighing the material immediately after sampling, drying at 105 °C, and reweighing 24 h later; a process which is destructive, time-consuming and hard to be automated.

A growing attention in the scientific community has been directed to terahertz spectroscopic techniques, mostly time domain spectroscopy or confocal microscopy [12], as a non-invasive tool for measuring leaf water content [13, 14] and related quantities such as drought stress [15, 16] and dehydration kinetics [17]. The potential of THz absorption measurements was also demonstrated in the study of the hydration of biomolecules [18–20] and ions [21–24]. The terahertz spectral region is particularly promising in this branch of studies: thanks to the large absorption coefficient of water [25], opposite to the relatively small absorption coefficient of the leaf dry matter [13] terahertz techniques are more sensitive to changes in water content than near infrared and microwave techniques, because they suffer less disturbances from changes in concentration of soluble substances, such as inorganic salts [26]. Terahertz techniques have to face the major challenge of the translation from lab tables to the field. The measurement method must be thought of for non-ideal working conditions, hence robust and reliable, in addition to non-invasive [27].

In this work, we report on a method for measuring the leaf wet mass using a terahertz quantum cascade laser [28] (THz QCL) and a camera. This method is based on a linear law which relates the leaf wet mass to the product between its optical depth and projected area. We compared this new method with the one based on the sole terahertz transmission, by studying six plants of *Vitis vinifera* L. (cv “Colorino”). A linear regression model was employed to fit the data obtained from the two methods. In the first case we had the sole optical depth as function of the leaf wet mass, and the least square fit of the data set produced an adjusted coefficient of determination of 0.31. With the new method instead, the linear regression model was adopted to fit the product between the leaf optical depth and the leaf projective area, as function of the leaf wet mass. In this case, the least square fit of the dataset has a coefficient of determination of 0.95, which means that the new method is reliable also when leaves come from different plants.

## Methods

### Plant preparation

All the experiments were performed in September 2015 on 2 year old vines. Six plants were pruned as a single-cordon and grafted onto SO4 (*V.* berlandieri $\times$ *V.* riparia) rootstock. *Vitis vinifera* L. (cv “Colorino”) were used for the trials. The plants were arranged to 2 L pots filled with a peat:sand mix (2:1). The vines were acclimated to the same environmental condition (24 °C; $45.5\%$ of relative humidity; approximately $660\, \upmu \text {mol}\, \text {m}^{-2}\, \text {s}^{-1}$ P.A.R.) inside the laboratory of the Department of physics at the University of Pisa, Italy. The measurements were performed under the same environmental conditions. Photon Flux measurements were conducted using em50 data logger (Decagon Devices, Inc.) equipped with a calibrated QSO-S PAR Photon Flux sensor. The environmental conditions, temperature and humidity, were measured with a calibrated usb temperature and humidity data logger (model IMD 100, Imagintronix Inc.). During the experiments, the photoperiod was 13 h 15 min day and 10 h 45 min night. For each of six plants all leaves fully extended and developed were sampled from the main shoot and, according to Kapos [29], the water content of leaves was determined by weighing the leaves immediately after sampling, drying at 105 °C, and reweighing 24 h later.

### Measurement setup

**Fig. 1:**
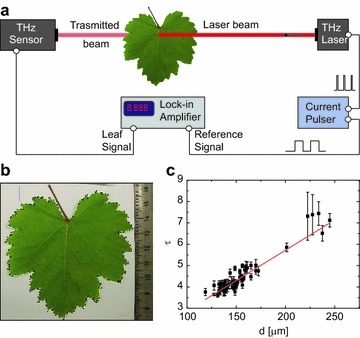
**a** Experimental setup for THz transmission measurements. A current pulser drives a THz cryo-cooled QCL to generate $2.5 \, \text {THz}$ laser radiation. The laser beam (*red straight line* in the picture) is coupled to a Fresnel Lens and a Picarin lens to produce a $8\, \text {mm}$ spot on the plane where the leaf is placed. The leaf is still attached to the plant, and its position is manually controlled. The laser beam transmitted by the leaf is collected by a Golay cell and sent to a lock-in amplifier. **b** Example of leaf projected area measurement. The picture to be analyzed is made by the fresh leaf with its *upper side* facing down on a *white sheet*, and a reference scale (ruler on the *right hand side*). Fiji ImageJ segmenting line tool was employed to trace the leaf contour and measure the area of the closed figure by using the reference scale. **c** Measured optical depth as function of the leaf thickness. The experimental data (*black squares*) are linearly fitted (*red straight line*), according to the Lambert–Beer’s law in Eq. (4). The linear fit has a coefficient of determination $R^2 = 0.77$; the slope $\alpha = 280 \pm 10\, \text {cm}^{-1}$ stems from the absorption coefficient $\alpha$ of the material, and it is consistent with other absorption coefficients measured for other plant species [16]. The model intercept $C_0 = 0.05 \pm 0.15$ has a *p* value of 0.75. Its poor statistical significance enforces the assumption of a Lambert–Beer’s law

Leaf optical depths were measured by means of a simple transmission setup, like the one sketched in Fig. 1a. THz laser radiation produced by a THz QCL is shined onto the leaf. The fraction of light passing through the leaf tissues is then collected by a THz detector. The ratio between the total incident power and the transmitted one gives the experimental optical transmission data. The optical source was a THz QCL emitting at frequencies around 2.5 THz, cryo-cooled down to 30 K and driven by a current pulser. For each leaf, we measured its transmittance and thickness; then, right after detachment, leaf fresh mass and projective area were measured. In order to sample most of the leaf structure, the transmittance data were sampled over four different regions with a diameter of 8 mm: one from the petiolar sinus to the first bifurcation; one on the first order vase in the distal part; two on the lateral lobes, left and right. The regions are loosely defined in order to assess the robustness of the measurement procedure when different leaves are measured. The readout signal is measured by a Stanford SR830 lock-in amplifier synchronized with the QCL current supply. To further suppress unwanted background and residual scattered radiation on the detector, a black screen with a 8 mm hole was placed behind the leaf sampling region. The black screen was made of an aluminum plate with a rough surface. In order to choose the hole width, a lever iris was placed along the optical path (the aluminum plate was removed) and the signal recorded by the detector was measured with increasing iris apertures, until the recorded signal did not change its least significant digit with respect to the case without the iris. During the transmission measurement the sample was held by hand. At the cost of some precision, the hand offers many translational and rotational degrees of freedom, enabling hardly reachable leaves to be placed in the optical path. For each leaf the average optical depth was calculated starting from the transmittance data, by averaging over the four samples. The leaf thickness $d_j$ was measured with a contact caliper by averaging over four $6\,\text {mm}$ diameter regions, selected with the same criteria as the regions for the transmission measurement. Note that the thickness was measured over a region without major veins; in this way the thickness is underestimated by a factor $5\text {--}10\%$ according to the literature [30]. The plant material was then detached and weighted to acquire the leaf fresh mass. Each projective area was measured immediately after weighting, with a procedure explained in Fig. 1b. The leaf was placed with its lower side facing up onto a white paper sheet with a scale reference on a side, and photographed. Its projective area was measured using Fiji ImageJ segmented line tool on the image collected [31]. Finally the leaf was dried for 24 h at a temperature of 105 °C and weighted to acquire its dry mass.

## Results

The main scope of our work is to find a relation between the leaf optical properties at THz frequencies and the leaf water content, which is reproducible for different plants of the same variety. We examined six plants of *Vitis vinifera* L. (cv “Colorino”) by measuring 58 leaves, chosen according to the Plant Preparation Section. The optical transmittance was sampled over four regions for each leaf, according to the Measurement setup Section. Given the j-th leaf, the optical transmittance of the k-th sample region is defined as the ratio between the laser beam intensity transmitted by the leaf to the detector, $S_{j,k}$, and the incident one, $I_j$:

1 $$\begin{aligned} T_{j,k} = \frac{S_{j,k}}{I_j}\text {.} \end{aligned}$$

In order to perform statistical data analysis, for each leaf we calculate its average experimental optical depth $\tau _{j,k}$, defined by the Lambert–Beer’s law [32]

2 $$\begin{aligned} T_{j,k} = \exp {[-\tau _{j,k}]}, \end{aligned}$$

therefore $\tau _{j,k}$ accounts for material losses. The average optical depth $\tau _j$ can be calculated as

3 $$\begin{aligned} \tau _{j} = \frac{1}{4}\sum _{k=1}^{4}{\tau _{j,k}}. \end{aligned}$$

Within an effective medium approximation [33, 34], $\tau _{j}$ is proportional to the effective leaf absorption coefficient $\alpha _j$ and the leaf average thickness $d_j$:

4 $$\begin{aligned} \tau _{j} = \alpha _j d_{j}. \end{aligned}$$

If all the leaves had the same distribution of water, air, and dry materials, their $\alpha _j$ should be the same. In Fig. 1c all the measured $\tau _j$ are plotted against $d_j$, each leaf contributing with one point. The red straight line reports the linear best fit, resulting from a two parameter linear regression in which data are statistically weighted with the inverse of their variances, to account for heteroscedasticity. We found that our linear regression has a coefficient of determination of $R^2 = 0.77$, which means it is able to explain $77\%$ of the overall $\tau _j$ fluctuations around their mean value; the resulting effective absorption coefficient $\alpha = 280 \pm 10 \, \text {cm}^{-1}$ is similar to the ones found for other species (for example, measured *Coffea Arabica* effective absorption coefficient is $\alpha \simeq 250 \, \text {cm}^{-1}$ at a frequency of $1.8 \, \text {THz}$ [16]). Also the high *p* value related to the intercept parameter ($C_0$ in Fig. 1c) confirms the consistency of the assumed Lambert–Beer’s law. It is worth to point out that we chose not to report the *p* value of those parameters having *p* value smaller than $10^{-10}$.

### Leaf water content and optical depth

**Fig. 2:**
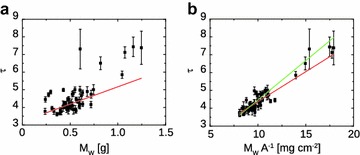
**a** Measured optical depth $\tau$ as function of the leaf water mass $M_W$. The experimental data (*black squares*) are linearly fitted (*red straight line*) as $\tau = C_1 + C_2 \cdot M_W$. The linear fit has a coefficient of determination $R^2 = 0.31$; the coefficients are $C_1 = 3.2 \pm 0.1$ and $C_2 = 2.0 \pm 0.1 \, \text {g}^{-1}$. **b** Measured optical depth versus $M_{W} A^{-1}$. The coefficient of determination is greatly improved with respect to the previous model, by the simple measurement of the leaf projective area *A*. In this case the linear model produces a best fit with $R^2 = 0.74$. There are two statistically significant parameters: the intercept $C_1 = 1.0 \pm 0.1$ may be ascribed to residual scattering and absorption from the leaf dry mass and vapor, whereas the slope $K = 0.33 \pm 0.03 \, \text {cm}^2 \, \text {mg}^{-1}$ is linked to the effective absorption coefficient of water; according to Eqs. (5) and (6) $\alpha _{\text {eff}} = 330 \pm 30 \, \text {cm}^{-1}$. If the intercept is set to 0 as proposed in Eq. (6), the absorption coefficient changes to $\alpha _{\text {eff}} = 444 \pm 2 \, \text {cm}^{-1}$, and the model fit accuracy is reduced (see the *green line in the graph*)

Trying to extract the leaf water mass from global optical depth is not trivial, because air, water and dry materials might be combined in different fractions and give approximately the same absorption coefficient. This concept is graphically explained in Fig. 2a, where the experimental $\tau$ has been plotted as function of the experimental leaf water mass $M_W$, which was measured for each leaf according to the Mesurement Setup section. In this case the linear slope is statistically different from 0 with *p* value of $3.8e-6$, but the linear regression explains only $31\%$ of the total data fluctuations. Better effective medium models could improve the predictions on leaf water mass. Recently, Landau–Lifshitz–Looyenga model has been successfully employed on *Coffea Arabica* and *Hordeum Vulgare* plants [13, 16], at the expense of new variables to be measured, such as the leaf dry material refractive index, which requires the leaf to be dehydrated and pelletized. In this work we found a simpler model to enhance the description of the experimental data, by combining $M_W$ with the leaf projective area *A*. If the optical depth is dominated by water absorption, Eq. (4) may be rewritten as:

5 $$\begin{aligned} \tau = \alpha _{\text {eff}} d_{W}, \end{aligned}$$

where $\alpha _{\text {eff}}$ is the effective absorption coefficient and $d_W = V_W/A$ is the effective water thickness inside the leaf, expressed as the ratio between the volume of water inside the leaf and the leaf projective area. Water volume may be safely expressed as function of the water mass $M_W$, because water density is known and can be approximated to $\rho _W = 1000 \, \text {mg cm}^{-3}$; Eq. (5) then becomes

6 $$\begin{aligned} \tau = \mathbb {K} \frac{M_W}{A}\,\text {,} \end{aligned}$$

where $\mathbb {K} = \alpha _{\text {eff}}/\rho _W$. Looking at Fig. 2b, we see the linear regression of $\tau$ as function of $M_W \cdot A^{-1}$. In this case the best fit of linear regression (red line) returns $\mathbb {K} = 0.33 \pm 0.03 \, \text {cm}^2 \, \text {mg}^{-1}$, which means an absorption coefficient $\alpha _{\text {eff}} = 330 \pm 30 \, \text {cm}^{-1}$, but also an intercept $C_1 = 1.0 \pm 0.1$, wich is statistically different from 0 with very small *p* value; this result entails a non-negligible contribution to losses from dry mass and/or vapor absorption, and from scattering due to the heterogeneity of the leaf tissues. The coefficient of determination is greatly improved with respect to the sole use of $M_W$, with $R^2 = 0.74$, which means that optical depth data describe almost $75\%$ of the $M_W \cdot A^{-1}$ data fluctuations. If the intercept is set to 0, from the linear fit (green line in Fig. 2b) we find $\alpha _{\text {eff}} = 444 \pm 2 \, \text {cm}^{-1}$; in this case the accuracy of the model is slightly reduced, because the residual sum of squares $\sum _{j=1}^{58} (\tau _j - \tau _j(\text {model}))$ increases by ${\sim }29\%$. By adding a term which is inversely proportional to *A*, Eq. (6) is transformed into

7 $$\begin{aligned} \tau = \frac{C_1}{A} + C_2 \frac{M_W}{A} \, \text {,} \end{aligned}$$

where $C_1$ and $C_2$ are constant coefficients. By moving *A* on the left hand side of the equation we obtain a linear relation

8 $$\begin{aligned} \tau A = C_1 + C_2 M_W. \end{aligned}$$

**Fig. 3:**
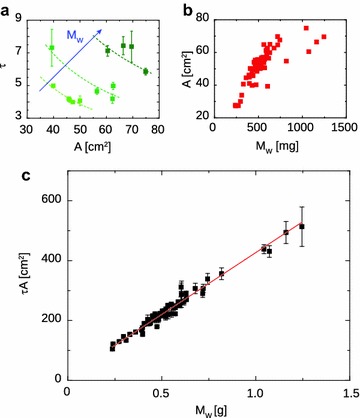
How the nonlinear relations between *A*, $\tau$ and $M_W$ can be used to improve the linear regression model. **a** Measured $\tau$ versus *A*. We report only some of the experimental data points, for the sake of clarity, grouped in three set according to similar mass: the *light green squares* represent all the samples having $M_W = 430 \pm 10$ mg, the *emerald squares* $M_W = 610 \pm 10$ mg, and the *dark green squares* $M_W = 1150 \pm 100$ mg. The *dashed curves* are obtained from Eq. (6), using the mean $M_W$ of each data group. **b** Measured *A* versus $M_W$. Referring to the data points of Fig. 2b, multiplication of $\tau$ and $M_{W} A^{-1}$ by *A* improves the linearity of the model. The specific nonlinear relation between *A* and $M_W$ (**b**) spreads the data cloud horizontally, whereas the inverse proportionality between $\tau$ and *A* (**a**) reduces the data fluctuations at given $M_w$. **c** The process explained before results in the graph of $\tau A$ as function of the water mass $M_W$. In this case the linear best fit (*red line*) has two statistically significant parameters. The intercept $C_1 = 14 \pm 3 \, \text {cm}^2$ (*p* value $8.1 \times 10^{-7}$) is ascribed to light scattering, whereas the slope $C_2 = 413 \pm 6 \, \text {cm}^2 \, \text {g}^{-1}$ may be still related to an effective absorption coefficient of water. The linear regression best fit has a coefficient of determination $R^2 = 0.95$, which means that our linear model explains $95\%$ of the experimental data fluctuations

In Fig. 3c we report the linear regression best fit of the experimental data $\tau _j A_j$ as function of the leaf water mass data $M_j$. We found that the variable $\tau A$ describes the water mass experimental data with $95\%$ of accuracy, and both coefficients have statistically relevant values, $C_1 = 14 \pm 3 \, \text {cm}^2$ (*p* value $8.1 \times 10^{-7}$) and $C_2 = 413 \pm 6 \, \text {cm}^2 \, \text {g}^{-1}$. The inverse proportionality with *A* of the term $C_1 A^{-1}$ in Eq. (7) could be ascribed to the nonlinear correlation between the leaf projective area and the water mass, as shown in Fig. 3a (lower graph): the larger is $M_W$, the slower is the increase of *A*, and the contribution of bulk absorption becomes more important.

## Discussions

The reason why the use of Eq. (8) improves the linear regression, as shown in Fig. 3c, can be found inside the nonlinear relation between *A*, $M_W$, and $\tau$, and it is graphically explained in Fig. 3, panels a and b. Here we report the two graphs of $\tau$ versus *A* (panel a) and *A* versus $M_W$ (panel b). Multiplication by *A* on both sides of Eq. (7) produces two synergical actions. It has a horizontal action, because lower values of *A* grow more steeply with $M_W$ than higher values, as shown in Fig. 3b; this effect tends to horizontally elongate the data cloud in Fig. 2b. There is also a vertical action, which can be explained looking at Fig. 3a. Here we reported three groups of data points, based on similarity of $M_W$. The first group (light green squares) has $M_W = 430 \pm 10$ mg, the second (emerald squares) $M_W = 610 \pm 10$ mg, the third (dark green squares) $M_W = 1150 \pm 100$ mg. The dashed lines indicate the curves found using Eq. (6) and the mean $M_W$ of each group. Since the curves reasonably fit the data points, we may consider *A* and $\tau$ to be inversely proportional; consequently, multiplication of $\tau$ by *A* reduces the data fluctuations at given $M_W$. The good agreement between the model Eq. (8) and the experimental data tells us that the model is robust enough to describe different plants of the same variety. During our experiment, leaves were detached from the plants after measuring the THz transmission, in order to perform the gravimetric measurements. Our treatment provided the leaf projective area by simply placing the leaf onto a white page with a scale bar on it. This method can of course be improved by placing a scalebar directly in the THz transmission setup. With this simple adaptation, the measurement of both the optical depth and the leaf projective area would become telemetric, thus exploitable for non-invasive measurements. Two other important points to take care of are the cryogenic temperature of the THz source and the leaf temperature. The cryo-cooler employed in this work mounts a Gifford-McMahon motor, which is free from cryogenic liquids such as liquid helium or liquid nitrogen; despite that, its size and weight would be impractical for in field measurements, requiring relatively large water-cooled compressors. This issue can be overcome by using low-current, high-efficiency THz QCLs [35]: they can be operated on board of compact, self-contained Stirling cryo-coolers, that necessitate only of limited electrical power (${\sim }10$ W) and air cooling. Temperature is also an important factor in determining the water absorption coefficient, whose variation is of the order of $10\%$ ranging from 25 to 35 °C. In this case a model describing how the dielectric permittivity of water changes with temperature is discussed in [36]: after the absorption coefficient is defined as function of the temperature, a conversion table can be arranged in order to normalize the in-field data with respect to the leaf temperature conditions, which could be probed for example by using an infrared thermometer [37]. Finally, our results unlock an interesting question about the measurements. The confidence of our linear fit depends on the specific relation between leaf water mass and leaf projective area. This relation could change with the plant species, and it will be the subject of future theoretical and experimental studies. The main result of this work can now be summarized: using Eq. (8) we are able to measure the water mass inside a grapevine leaf by means of two potentially telemetric measurements, which are fast, non-invasive, and reliable.

## Conclusions

In this work, we developed a new method for measuring the leaf water mass using a THz QCL, based on a leaf statistical sampling and a linear equation which relates the leaf water mass to the product between the optical depth and the leaf projected area. The results are in very good agreement with the model proposed here, proving the robustness and reliability of our new technique, which is under patent filing (application no. 102016000106179). Our method can be employed when plant averaged or cumulative data are required. For example, the canopy water mass can be combined with other plant parameters such as the biomass fresh and dry weight or the grain yield, and used as diagnostic indicators for cultivars under drought stress [38]. In future experiments, further calibration curves will be performed on other plant genotypes in order to prove our law or find other ones, whereas new relations between the shape of the scaling law and different plant behaviours could be highlighted, e.g. when preserving the water status during drought stress. By combining our technique with a gas exchange analyser, experiments can be carried out monitoring plant physiological aspects such as stomatal conductance and water potential directly in the laboratory or in the field. Furthermore, the different vein architecture among polyhedral leaves may be related to different management of water demand within the hydraulic network.
